# Bronchial asthma is associated with increased risk of chronic kidney disease

**DOI:** 10.1186/1471-2466-14-80

**Published:** 2014-05-08

**Authors:** Hui-Ling Huang, Shinn-Ying Ho, Chien-Hsun Li, Fang-Ying Chu, Li-Ping Ciou, Hua-Chin Lee, Wen-Liang Chen, Nian-Sheng Tzeng

**Affiliations:** 1 Institute of Bioinformatics and Systems Biology, National Chiao Tung University, Hsinchu, Taiwan; 2 Department of Biological Science and Technology, National Chiao Tung University, Hsinchu, Taiwan; 3 Department of Surgery, Division of Neurosurgery, National Taiwan University Hospital Hsin-Chu Branch, Hsinchu City, Taiwan; 4 Department of Psychiatry, Tri-Service General Hospital, School of Medicine and Student Counseling Center, National Defense Medical Center, #325, Sec 2, Cheng-Gong RdNei- Hu District, Taipei City, Taiwan

**Keywords:** Bronchial asthma, Chronic kidney diseases, National Health Insurance Research Dataset

## Abstract

**Background:**

Bronchial asthma influences some chronic diseases such as coronary heart disease, diabetes mellitus, and hypertension, but the impact of asthma on vital diseases such as chronic kidney disease is not yet verified. This study aims to clarify the association between bronchial asthma and the risk of developing chronic kidney disease.

**Methods:**

The National Health Research Institute provided a database of one million random subjects for the study. A random sample of 141 064 patients aged ≥18 years without a history of kidney disease was obtained from the database. Among them, there were 35 086 with bronchial asthma and 105 258 without asthma matched for sex and age for a ration of 1:3. After adjusting for confounding risk factors, a Cox proportional hazards model was used to compare the risk of developing chronic kidney disease during a three-year follow-up period.

**Results:**

Of the subjects with asthma, 2 196 (6.26%) developed chronic kidney disease compared to 4 120 (3.91%) of the control subjects. Cox proportional hazards regression analysis revealed that subjects with asthma were more likely to develop chronic kidney disease (hazard ratio [HR]: 1.56; 95% CI: 1.48-1.64; *p* < 0.001). After adjusting for sex, age, monthly income, urbanization level, geographic region, diabetes mellitus, hypertension, hyperlipidemia, and steroid use, the HR for asthma patients was 1.40 (95% CI: 1.33-1.48; *p* = 0.040). There was decreased HRs in steroid use (HR: 0.56; 95% CI: 0.62-0.61; *p* < 0.001) in the development of chronic kidney disease. Expectorants, bronchodilators, anti-muscarinic agents, airway smooth muscle relaxants, and leukotriene receptor antagonists may also be beneficial in attenuating the risk of chronic kidney disease.

**Conclusions:**

Patients with bronchial asthma may have increased risk of developing chronic kidney disease. The use of steroids or non-steroidal drugs in the treatment of asthma may attenuate this risk.

## Background

Bronchial asthma is a chronic inflammatory lung disease with exacerbations, which may be a factor in its morbidity and mortality. The Global Initiative for Asthma (GINA) 2004 report states that nearly 300 million people suffer from asthma worldwide [1,2] and asthmatic patients in Taiwan account for 2.6% [1]. The report also states that urban living [3] and lower income [1] are risk factors for asthma and that the environment, regardless of indoor or outdoor, also impacts on patients with asthma [3].

Bronchial asthma also influences other chronic diseases involving the cardiovascular and carbohydrate metabolism systems. Patients with asthma have higher risks of coronary heart disease (CHD) [4], diabetes mellitus [5], and hypertension [3], although the impact of asthma on other vital organs are not yet verified.

Chronic kidney disease (CKD) is a major global problem. Patients who progress to end-stage renal disease (ESRD) need dialysis or transplantation, which cause heavy medico-economic burden. In 2010, there were 651 000 patients with ESRD in the United States whose care cost around US $28.3 billion. In Taiwan, there were approximately 11.93% with CKD, about one eighth for Taiwanese. Recent studies have found that predictors of CKD include metabolic syndrome [6], obesity [7], vascular diseases [8], hyperlipidemia [9] and cardiovascular diseases [10], hypertension [11], diabetes mellitus [11], and heart disease [12] (OR: 1.95 [1.32-2.87]). However, there is no report on the association between asthma and kidney diseases.

This study used the Taiwan National Health Insurance Research Database (NHIRD) to determine the association of asthma and CKD in a three-year follow-up period (2003–2007). The National Health Insurance (NHI) Program, began in Taiwan in 1995, had enrolled 22 928 190 people as of June 2009, exceeding 99% of the population. The NHI also has a contract with 97% of the medical providers in Taiwan [13]. The diagnostic coding used for the NHI in Taiwan is according to the International Classification of Diseases, 9th Revision, Clinical Modification (ICD-9-CM) diagnostic criteria [14]. Each diagnosis of asthma was made by board-certified internists, clinical immunologists, pulmonologists, or other medical experts. The Bureau of National Health Insurance randomly reviews the charts of 1 per 100 ambulatory and 1 per 20 in-patient claim cases to verify the accuracy of the diagnosis [15]. In Taiwan, the diagnosis of CKD follows the criteria of “Kidney Disease: Improving Global Outcomes (KDIGO)”. CKD is defined as kidney damage as albumin-to-creatinine ratio >30 mg/g in two of three spot urine specimens or glomerular filtration rate (GFR) <60 mL/min/1.73 m^2^ for 3 months or more, irrespective of the cause [16]. The diagnosis of bronchial asthma bases on characteristic clinical history such as intermittent breathlessness, wheezing, troublesome night time cough and chest tightness, aided by lung function tests in some cases [17,18], which is similar to criteria of the Global Initiative for Asthma (GINA) guidelines [19,20].

## Methods

### Population data

This research used the Longitudinal Health Insurance Database (LHID) 2005 derived from NHI program [21]. The LHID covered more than 25 million Taiwanese, or approximately 98% of the citizens [22] who lived in Taiwan more than four months.

The longitudinal database was a subset of the National Health Insurance Research Database (NHIRD) and consisted of 1 million Taiwanese enrolled in the database in 2005 [23]. It contained home care visits, ambulatory care, out-patient care, prescription drugs, and medical record that was encoded by using the International Classification of Disease, 9- revision, Clinical Modification (ICD-9-CM) [24].

The Internal Review Board encrypted the database to remove any personal identification before the release of the dataset for public access. In this study, both the study and control cohorts had no significant difference in sex and age distribution and were selected randomly from LHID2005. Patients aged ≥18 years old who were first diagnosed with asthma (ICD-9-CM: 493) between January 1, 2004 and the end of 2007 were included in the study cohort and their date of diagnosis was their index date. The control cohort consists of the patients having no history of bronchial asthma or CKD from 2000 to 2010. These patients were randomly chosen by matching the gender and age of the study cohort with a ratio of 1:3. In addition, the middle date of the same month with that of the index date in the study cohort was assigned to the index date of the corresponding patient in the control cohort.

The patients were followed-up for three years from the index date until the patient was diagnosed with CKD (ICD-9-CM: 580, 581, 582, 583, 584, 585, 586, 587, 588, 589, 753, 403, 404, 2504, 2741, 4401, 4421, 4473, 5724, 6421, and 6462) or until the end of 2010. Patients with missing data were excluded, as well as those with CKD before asthma. The final study and control cohorts had 35 086 and 105 258 patients, respectively. The follow-up interval of the subjects and controls for developing chronic kidney disease during is in a three-year follow-up period.

The covariates considered included diabetes mellitus (ICD-9-CM 250, 357.2, 362.0x, and 366.41), hypertension (ICD-9-CM 362.11, 401.x-405.x, and 437.2), hyperlipidemia (ICD-9-CM 272.x), heart disease (ICD-9-CM 410–429 and A codes A270, A279-A281, and A289) and obesity (ICD codes: 278.00, 278.01, 278.02, 278.03). Data on intake of steroids, anti-asthma drugs, and other drugs like expectorants, bronchodilators, anti-muscarinic agents, and airway smooth muscle relaxants, were collected.

### Ethical approval

The Institutional Review Board of Changhua Christian Hospital approved the study (IRB/CCH, No. 121007).

### Statistical analysis

The SPSS software version 19.0 (SPSS Inc., Chicago, IL, USA) was used for all statistical analyses. The chi-square test was used for the descriptive analyses concerning distribution of population, level of urbanization (i.e., provinces, counties, districts, and urban villages), geography (northern, central, southern, and eastern), income (<18000, between 18000 and 35000, and ≥35000), co-morbidities (e.g., diabetes, hypertension, hyperlipidemia, and heart disease) and medications.

Multiple logistic regression analyses were performed to estimate hazard ratio (HR) and 95% confidence intervals (CIs) of bronchial asthma associated with CKD after controlling for age, sex, levels of urbanization and income. Statistical significance was set at *p* < 0.05.

## Results

The asthma subjects (n = 35086) and non-asthmatic controls (n = 105258) both had a mean age of 47.72 years (Table 1). There was no significant difference in distributions of sex, age, urbanization, geography, and economic status between the two groups. The patients with asthma had higher prevalences of diabetes mellitus, hypertension, hyperlipidemia, heart disease and obesity compared to the controls. More than 50% of the asthma patients (n = 19720, 56.2%) used steroids as treatment for asthma.

**Table 1:** Distribution of sex, age, urbanization, geography, income, co-morbidity, and medication of individuals with asthma and without asthma

**Characteristic**	**No. (%) of individuals**		
	**With asthma n = 35086**	**Without asthma n = 105258**	
			***p *** **value**
Age, yr, mean (SD)	47.72 ± 17.73	47.72 ± 17.73	1
Sex			
Male	15460 (44.1%)	46380 (44.1%)	
Female	19626 (55.9%)	58878 (55.9%)	
Age Group			1
18-29	6534 (18.6%)	19602 (18.6%)	
30-39	6307 (18.0%)	18921 (18.0%)	
40-49	6411 (18.3%)	19233 (18.3%)	
50-59	5751 (16.4%)	17253 (16.4%)	
≥60	10083 (28.7%)	30249 (28.7%)	
Urbanization			<0.05
Provinces	8777 (25.0%)	28167 (26.8%)	
Counties	2568 (7.3%)	8464 (8.0%)	
Districts	8988 (25.6%)	26504 (25.2%)	
Urban villages	14753 (42.0%)	42123 (40.0%)	
Geography			<0.05
North	18559 (52.9%)	52460 (49.8%)	
Central	6502 (18.5%)	18653 (17.7%)	
South	9151 (26.1%)	31605 (30.0%)	
East	874 (2.5%)	2540 (2.4%)	
Income			<0.05
<18000	15880 (45.3%)	49785 (47.3%)	
18000-35000	14363 (40.9%)	40763 (38.7%)	
>35000	4843 (13.8%)	14710 (14.0%)	
Co-morbidity			
Diabetes	1457 (4.2%)	2811 (2.7%)	<0.05
Hypertension	2472 (7.0%)	4467 (4.2%)	<0.05
Hyperlipidemia	1629 (4.6%)	2983 (2.8%)	<0.05
Heart Disease	2115 (6.0%)	3152 (3.0%)	<0.05
Obesity	91 (0.3%)	105 (0.1%)	<0.05
Medication			
Steroids	19720 (56.2%)	24373 (23.2)	<0.05

**Figure 1:**
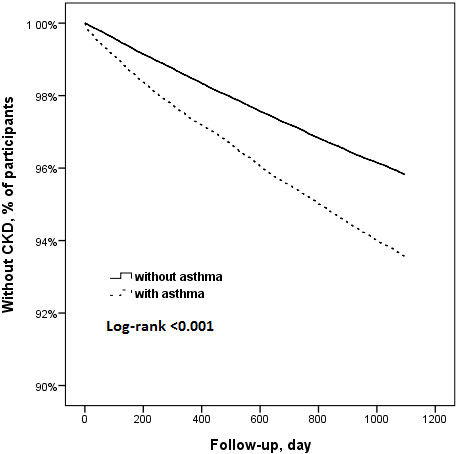
Survival curves of patients with and without asthma.

On Kaplan Meier (KM) survival curve, two years would have produced significant CKD (*p* < 0.001) (Figure 1). Thus, a follow-up time of three years would have been more suitable in this study.

Subjects with asthma (2196 in 35086) were more likely have CKD compared to controls (4125 in 105258) (HR: 1.56, 95% CI: 1.48-1.64; *p* < 0.001) (Table 2).

**Table 2:** Individuals with and without asthma as a predictor of chronic kidney disease, identified by Cox regression

**Characteristic**	**No. (%) of individuals**	
	**With asthma n = 35086**	**Without asthma n = 105258**
With chronic kidney disease	2196	4125
Without chronic kidney disease	32890	101106
Hazard ratio	1.56 (1.48-1.64)^‡^	

^‡^*p* < 0.001 for comparison between patients with asthma and the controls.

After adjusting for sex, age, andco-morbidities, including obesity, patients with asthma developed CKD (HR: 1.13 [1.07-1.19], *p* < 0.001) compared to HR before the adjustments (HR: 1.56 [1.48-1.64], *p* < 0.001) (Table 3). Male asthma patients were more likely to have CKD than female asthma patients (HR: 1.16 [1.11-1.22], *p* < 0.001). Patients with co-morbidities, including diabetes mellitus (HR: 1.42 [1.33-1.51], *p* < 0.001), hypertension (HR: 15.52 [14.18-16.99], *p* < 0.001), hyperlipidemia (HR = 1.55 [1.45-1.65], *p* < 0.001), and heart disease (HR: 1.86 [1.71-1.98], *p* < 0.001), had high risks of CKD. Hypertension was the co-morbidity with the highest risk of developing CKD. After adjusting for steroid treatment, the adjusted HR of developing CKD was 1.40 in asthma subjects. Patients who used steroids had lower risk of developing CKD (HR: 0.50 [0.47-0.53], *p* < 0.001).

**Table 3:** Independent predictors of chronic kidney disease, by Cox regression analysis

**Variable**	Adjusted HR* (95% CI)	Adjusted HR** (95% CI)
Asthma	1.13 (1.07-1.19)^‡^	1.40 (1.33-1.48)^‡^
Female	reference	reference
Male	1.16 (1.11-1.22)^‡^	1.16(1.10-1.22)‡
Age group		
18-29	reference	reference
30-39	1.51 (1.30-1.76)^‡^	1.52 (1.31-1.77)^‡^
40-49	1.91 (1.66-2.19)^‡^	1.99 (1.73-2.28)^‡^
50-59	2.02 (1.76-2.31)^‡^	2.14 (1.86-2.45)^‡^
≥60	2.08 (1.83-2.38)^‡^	2.23 (1.96-2.55)^‡^
Co-morbidity		
Diabetes	1.42 (1.33-1.51)^‡^	1.42 (1.33-1.52)^‡^
Hypertension	15.52 (14.18-16.99)^‡^	15.24 (13.92-16.68)^‡^
Hyperlipidemia	1.55 (1.45-1.65)^‡^	1.53 (1.43-1.64)^‡^
Heart Disease	1.84 (1.71-1.98)^‡^	1.92 (1.78-2.06)^‡^
Obesity	1.3 (1.07-1.58)^†^	1.39 (1.14-1.69)^‡^
Medication		
Steroid (used in 56.2% asthma subjects)		0.50(0.47-0.53)^‡^

Note: ^†^*p* < 0.05 for comparison between patients with asthma and without asthma; ^‡^*p* < 0.001 for comparison between patients with asthma and without asthma.

HR*, each variable was adjusted for every other variable listed except for medication.

HR**, each variable was adjusted for every other variable listed.

Asthma patients taking steroids were compared to patients not taking steroids (Table 4). In the subgroup with steroid treatment (n = 26236), 1038 (3.96%) developed CKD in the longitudinal follow-up within three years. In the subgroup without steroid treatment (n = 8850), 1158 (13.08%) developed CKD in the same follow-up period. The HR of the steroid treatment subgroup was 0.56 (95% CI: 0.522-0.61, *p* < 0.001) after adjusting for co-morbidities of diabetes mellitus, hypertension, hyperlipidemia, and heart disease. Steroid treatment attenuated the risk of developing CKD (HR: 0.56, 95% CI: 0.62-0.61; *p* < 0.001) after adjusting for co-morbidities (Table 5).

**Table 4:** Steroid use in patients with asthma as a predictor of chronic kidney disease, by Cox regression analysis

**Characteristics**	**No. (%) of individuals**	
	**With steroid use n = 19720**	**Without steroid use n = 15366**
With chronic kidney disease	1038	1158
Without chronic kidney disease	18682	14208
Hazard ratio	0.74 (0.70-0.78)^‡^	
Hazard ratio*	0.56 (0.53-0.589)^‡^	

Note: ^‡^*p* < 0.001 for comparison between patients who used steroids and those who did not.

Hazard Ratio*, Adjusted for diabetes, hypertension, hyperlipidemia, and heart disease.

**Table 5:** Hazard ratios (HRs) of asthma patients by drug used compared to patients without drug use

**Variables**	**n (%)**	**HR (95% CI)**	HR* (95% CI)	HR** (95% CI) With steroid	HR*** (95% CI) Without steroid	HR**** (95% CI)
**Steroid**						
Yes (n = 19720)	1038 (5.3%)	0.68 (0.62-0.73)^‡^	0.56 (0.52-0.61)^‡^	N/A	N/A	N/A
No (n = 15366)	1158 (7.5%)					
**Expectorant**						
Yes (n = 6818)	339 (5.0%)	0.74 (0.66-0.83)^‡^	0.59 (0.53-0.67)^‡^	0.63 (0.54-0.74)^‡^	0.65 (0.55-0.78)^‡^	0.64 (0.57-0.72)^‡^
No (n = 28268)	1857 (6.6%)					
**Bronchodilators**						
Yes (n = 19933)	1157 (5.8%)	0.84 (0.77-0.91)^‡^	0.67 (0.61-0.72)^‡^	0.78 (0.68-0.89)^‡^	0.78 (0.69-0.88)^‡^	0.78 (0.71-0.85)^‡^
No (n = 15153)	1039 (6.9%)					
**Anti-muscarinic agents**						
Yes (n = 4298)	264 (6.1%)	1.00 (0.88-1.14)	0.72 (0.63-0.82)^‡^	0.87 (0.75-1.01)	0.86 (0.64-1.14)	0.87 (0.76-0.99)^‡^
No (n = 30788)	1932 (6.3%)					
**Airway smooth relaxant**						
Yes (n = 19222)	1245 (6.5%)	1.07 (0.99-1.17)	0.75 (0.69-0.82)^‡^	0.88 (0.77-1.02)	0.91 (0.81-1.03)	0.90 (0.82-0.99)^†^
No (n = 15864)	951 (6.0%)					
**Leukotriene receptor antagonist**						
Yes (n = 1432)	46 (3.2%)	0.48 (0.36-0.65)^‡^	0.59 (0.44-0.79)^‡^	0.72 (0.52-1.00)	0.62 (0.32-1.20)	0.69 (0.52-0.93)^†^
No (n = 33654)	2150 (6.4%)					

Note: HR*, each factor was adjusted for diabetes, hypertension, hyperlipidemia, and heart disease.

CI, confidence interval; n (%), number of participants with CKD and% of participants.

^†^*p* < 0.05 for comparison between patients with asthma and without asthma; ^‡^*p* < 0.001 for comparison between patient with asthma and without asthma.

Hazard ratios (HRs) of asthma patients by drug used. In each drug, the HR was compared with patients without drug. (a) Crude HR (95% CI) for each drug. (b) HR *(95% CI) for each drug, adjusted for co-morbidities. (c) Patients with steroid HR **(95% CI) for each drug, adjusted for co-morbidities. (d) Patients without steroid use HR ***(95% CI) for each drug, adjusted for co-morbidities. (e) HR **** (95% CI) for each drug, adjusted for co-morbidities and steroid use.

Non-steroidal drugs for treatment of asthma were grouped into five classes: expectorants, bronchodilators, anti-muscarinic agents, airway smooth muscle relaxants, and leukotriene receptor antagonists. After adjusting for co-morbidities, all of these drugs attenuated the HRs of developing CKD. Regardless of adjustments for co-morbidities and steroid use, expectorants and bronchodilators attenuated the risk for developing CKD.

## Discussion

This prospective case control study analyzed the relationship between asthma and CKD (HR: 1.56 [1.48-1.64], *p* < 0.001) using population-based data (LHID) in Taiwan. In the dataset, patients first diagnosed with asthma and without CKD between 2000 and 2003 were analyzed. The Kaplan-Meier survival curve showed a time-to-event of two years, such that patients with asthma would develop CKD in around two years (*p* < 0.001). As such, a follow-up time of three years was a sufficient follow-up period for this study.

Most researches report that patients with older age have increased risk of developing CKD [25]. Moreover, patients with hypertension [3], heart disease [4], diabetes [5], hyperlipidemia [9] and obesity [26-28] also have high risk of CKD. However, after adjusting for sex, age, and these co-morbidities, subjects with asthma still have significant and independent high risk of CKD (HR: 1.13 [1.07-1.19], *p* < 0.001).

Except for some rare case reports [29,30], there are very few studies investigating the interactions between asthma and CKD. To date, this is the first study to show that asthma patients are prone to developing CKD in comparison to non-asthma patients. Some patients with exercise-induce asthma seldom exercise [31] and several researches report that aerobic exercise may improve CKD [32,33]. Limited exercise may be one of the reasons for the higher risk of CKD in some types of asthma from the perspective of health behaviours.

Recently, one study of 2354 asthma patients from a retrospective cohort in China indicates that there is 9.6% incidence of CKD in a period of six-year follow-up, in which the group of persistent asthma has independent, higher risk of CKD than the non-persistent group. In addition, patients with three traits together of long history of asthma > 20 years, having no well-controlled asthma and persistent stage of asthma, have significant risk as high as OR = 3.39 (95% CI 1.36–8.73), compared to patients without these traits [34]. This study finds an association between asthma and later CKD using a large cohort comprising asthma subjects (n = 35086) and non-asthma controls (N = 105258) in a three-year period of follow-up. Steroid and other medication treatment might decrease the risk of CKD associated with asthma. The suggestion of the two studies is similar that the value of well-controlled asthma is high not only to preserve the respiratory function but also to benefit other organs.

Furthermore, one common pathophysiology, inflammation, may provide some preliminary explanations. Recent advances in the study of leucine-rich repeat protein 3 (NLRP3) inflammasome may also provide a potential link between asthma and CKD. The NLRP3 inflammasome contributes to the progression of CKD by promoting renal inflammation [35] and plays a role in the human rhinovirus-related primary bronchial cell inflammation [36].

In our study, the recruited subjects with asthma are in older age as 47.72 ± 17.73 years. Several studies showed that asthma could develop at age after childhood, adolescence or young adult stage, and several genetic factors, air pollution or obesity could be related to later onset of bronchial asthma [37-39]. One study of 504 asthmatic patients (303 males and 201 females) in Taiwan indicates that 29% developed asthma at age 25–44 years old, 21% developed asthma at age 45–65 years old, and 8% developed asthma beyond age of 64 [40]. The recent study indicates that the age of onset asthma is 45.4 ± 10.4 years, which is also similar to our study of 47.72 ± 17.73 years [34].

Aside from co-morbidities, drugs used in the treatment of asthma also influence the development of CKD. These include steroids and non-steroidal anti-asthma drugs. In this study, 56.2% of patients with asthma take steroids. Steroid use is associated with lower risk of CKD after adjusting for sex, age, and co-morbidities. Steroids can decrease inflammatory reactions and are used to treat inflammatory diseases. After adjusting for steroid use, the HR of asthma increased, indicating that asthma directly impacts on CKD. This also supports the hypothesis that chronic inflammation in asthma patients may be related to the higher risk of developing CKD. Proper steroid treatment for asthma may significantly reduce the risk of CKD.

Non-steroidal drugs used for asthma were also analyzed. In this study cohort, only patients with asthma were included. Classes of non-steroidal drugs were expectorants, bronchodilators, anti-muscarinic agents, airway smooth muscle relaxants, and leukotriene receptor inhibitors. After adjusting for diabetes, hypertension, hyperlipidemia, and heart disease, all of these non-steroidal drugs could attenuate the development of CKD. Nonetheless, regardless of adjustments for co-morbidities or steroid use, expectorants and bronchodilators can attenuate the risk for CKD. Although leukotriene receptors have anti-inflammatory properties, they have low potential of attenuating the risk for CKD [41]. However, the biological mechanisms involved warrant further investigations.

This study has some limitations. Although the LHID 2005 in Taiwan has benefits in analyzing disease and is commonly used in research because of its reliability, it has some limitations. First, the selected data might exclude samples because of missing data, making the data here incomplete. Second, the diagnostic code is made by doctors. However, sometimes the code is not final, especially in ambulatory settings. Third, the presence of diseases was base on ICD-9-CM. The precision of this system and its impact on outcome could not be determined. Furthermore, no information about cigarette smoking, which is a risk factor of CKD [42], is available in the NHIRD database. However, we have adjusted other metabolic risk factors such as diabetes mellitus, hypertension, hyperlipidemia, and obesity.

## Conclusions

Asthma patients have higher risk of developing CKD. Although interactions between asthma and CKD are still unclear, behavioural or biological factors such as limited exercise, inflammation, and other unknown factors can contribute to the interactions between asthma and kidney diseases. Steroids and some non-steroidal anti-asthma drugs can attenuate this risk and the proper medical treatment for asthma may be beneficial in reducing the risk of CKD.

## Competing interests

The authors declare that they have no competing interests.

## Authors’ contributions

NST conceived of the study, participated in its design and coordination, data interpretation and drafted the manuscript. HLH participated in the design of the study, data interpretation, performed the statistical analysis, data collection and drafting of the manuscript. SYH participated in the design of the study and participated in data interpretation. CHL participated in the design of the study, data interpretation and drafting of the manuscript. FYC participated in the design of the study, literature search, data collection, and performed the statistical analysis. LPC participated in data collection and data interpretation. HCL and WLC participated in the design of the study and data interpretation. All authors have read and approved the final manuscript.

## Pre-publication history

The pre-publication history for this paper can be accessed here:

http://www.biomedcentral.com/1471-2466/14/80/prepub
